# Long-Term Effects of Zinc Deficiency and Zinc Supplementation on Developmental Seizure-Induced Brain Damage and the Underlying GPR39/ZnT-3 and MBP Expression in the Hippocampus

**DOI:** 10.3389/fnins.2019.00920

**Published:** 2019-09-04

**Authors:** Ni-Na Chen, Dong-Jing Zhao, Yu-Xiao Sun, Dan-Dan Wang, Hong Ni

**Affiliations:** Division of Brain Science, Institute of Pediatric Research, Children’s Hospital of Soochow University, Suzhou, China

**Keywords:** zinc diet, developmental seizure, GPR39, ZnT-3, MBP, plasma membrane integrity

## Abstract

We previously illustrated that long-term upregulated expression of ZnT-3 in the hippocampus of rats that underwent neonatal seizures was restored by pretreatment with a ketogenic diet. It was recently demonstrated that upregulated expression of ZnT-3 was associated with increased concentrations of intracellular free zinc ions in an *in vitro* model of glutamate-induced hippocampal neuronal excitotoxic damage. However, there is still a lack of research on the effects of different concentrations of zinc in the diet on developmental convulsive brain injury. The aim of this study was to investigate the effects of different zinc concentrations in the diet on long-term neurobehavioral and seizure thresholds following lithium chloride-pilocarpine-induced developmental seizures. Sprague-Dawley rats (postnatal day 27, P27) were randomly assigned to one of six dietary groups for 4 weeks: normal zinc control group (Control group, 44 mg/kg Zn), Zn-deficient control group (ZD group, 2.7 mg/kg Zn), Zn supplemented control group (ZS group, 246 mg/kg Zn), pilocarpine-induced seizure plus regular zinc diet group (SE group, 44 mg/kg Zn), seizure plus low-zinc diet group (SE + ZD group, 2.7 mg/kg Zn), and seizure plus high-zinc diet group (SE + ZS group, 246 mg/kg Zn). Novel object recognition and passive avoidance tests were performed on rats at P42 and P56. After routine seizure threshold detection and Timm staining procedures at P57, expression of GPR39, ZnT-3, and MBP were detected in the hippocampus by Western blot analysis. The results revealed that the Zinc-deficient diet for 4 weeks aggravated the long-term adverse effects of developmental seizures, evidenced by weight, cognition, seizure threshold and serum zinc concentrations, which were paralleled by expression changes in hippocampal GPR39 and ZnT-3. In contrast, zinc supplementation for 4 weeks significantly improved damage-related changes described above and rescued the abnormal expression of GPR39, ZnT-3, and MBP in the hippocampus. Similar alterations between the expression pattern of MBP and aberrant sprouting of mossy fibers in the hippocampus may indicate that sprouting is a secondary pathological change caused by developmental brain damage rather than the cause of epileptogenesis. Up-regulation of MBP protein levels in the high zinc diet-treated seizure group as well as the corresponding improvement of cognitive impairment and reduced hippocampal mossy fiber regenerative sprouting, may represent a compensatory mechanism for neuronal membrane damage and repair.

## Introduction

Developmental seizure-induced brain damage is often accompanied by cognitive deficits, memory disorders, emotional abnormalities, etc. (Rzezak et al., 2017; Vidaurre and Twanow, 2017; De Toffol et al., 2018), which has serious consequences on families and society. At present, 20 to 30% of patients with epilepsy do not experience relief after reasonable standard drug treatment, which is known as refractory epilepsy (López González et al., 2015). Temporal lobe epilepsy is one of the most common forms of refractory epilepsy and is caused by changes in molecular networks, and structure and function after brain injury, leading to a pathological environment that ultimately promotes excitability and produces repeated spontaneous seizures. This process is known as epileptogenesis (Jehi and Vezzani, 2014). The mechanism of epileptogenesis may be multifactorial and is not yet fully understood, limiting the development of new drugs. Therefore, exploring the pathophysiological mechanisms of epileptogenesis is critical to the development of new, safe, and effective drugs.

An imbalance between neuronal excitation and inhibition defines the mechanism of epilepsy (Staley, 2015), and zinc ions are involved in convulsive excitotoxic neuronal injury. Zinc ions interact with various targets that mediate neuronal excitation or inhibition. For example, using zinc ion chelating agent (TPEN) to induce zinc deficiency in an *in vitro* neuronal cell model, zinc deficiency was shown to cause decreased cell viability and increased rates of apoptotic. These changes are reversed by zinc supplementation (Tian et al., 2018).

Zinc transporter 3 (ZnT-3) knockout mice are more sensitive to seizures caused by kainic acid injection due to their lack of synaptic zinc ions (Mcallister and Dyck, 2017), indicating that a deficiency in synaptic zinc ions reduces seizure thresholds. Ketogenic diet (KD) is a nutritional treatment that is beneficial in epilepsy refractory to antiepileptic drugs. It was previously shown that the mechanism(s) of KD’s action involve altered zinc metabolism, as KD rescues seizure-induced elevated ZnT-3 expression in the hippocampus (Tian et al., 2015). We recently demonstrated that glutamate stimulation of HT22 hippocampal neurons significantly increases intracellular zinc ion concentrations, which is positively correlated with mitophagy levels and mitochondrial dysfunction (Jin et al., 2018). These studies highlight the possibility that zinc ion signaling is a novel target for inhibiting epileptogenesis.

At present, there are few studies using *in vivo* models to investigate the effects of zinc intervention on epilepsy, and the results are often contradictory due to the type of epilepsy, the dose of zinc intervention and the route of administration. Kumar et al. (2015) once investigated the effect of zinc ions on acute seizures. They found that oral administration of 2, 20, or 200 mg/kg zinc sulfate for 2 weeks did not affect acute seizures induced by maximum electroconvulsive shock; however, 2 mg/kg zinc administration significantly reduced seizure duration and increased the latency of seizures induced by pentylenetetrazol (PTZ). In addition, 200 mg/kg zinc sulfate intervention significantly reduced the number of ignited animals and reduced the seizure severity score. In contrast, Baraka et al. (2012) reported the opposite results. They found that intraperitoneal injection of zinc sulfate at 60 mg/kg for 3 weeks increased the severity of pilocarpine-induced seizures. Therefore, it is necessary to further study the role of zinc in epilepsy and its underlying molecular mechanisms using *in vivo* animal models.

Assessing the effects of different concentrations of zinc diet on developmental seizure-induced brain damage may be an important step in elucidating the role of zinc in epilepsy. Based on the *in vivo* animal model of developmental seizures induced by lithium chloride-pilocarpine, this study explored the long-term effects of zinc deficiency and zinc supplementation on developmental seizure-induced brain damage, focusing on the parameters of cognition, seizure threshold, hippocampal regenerative mossy fiber sprouting and expression of ZnT-3 and GPR39 in hippocampus to further reveal the relationship between zinc and epileptogenesis and provide new insights for the prevention and treatment of epilepsy. G protein-coupled receptor 39 (GPR39) is a metabotropic zinc-specific receptor (Kovacs et al., 2014). GPR39 knockout enhances susceptibility to kainic acid-induced seizures and increases seizure duration (Gilad et al., 2015). In addition, we assessed expression of myelin basic protein (MBP) because it plays a key role in controlling neuronal membrane integrity and axonal regeneration (Snaidero et al., 2017).

## Materials and Methods

### Animal Preparation

Postnatal day 27 (P27) male Sprague-Dawley rats (*n* = 145) were obtained from the Zhao Yan (Suzhou) New Drug Research Center, Co., Ltd. [Animal License No. SCXK (Su) 2018-0006], China. Animals were treated in accordance with the guidelines set by the National Institutes of Health (Bethesda, MD, United States) for the humane treatment of animals and the Declaration of Helsinki. This study was approved by the Institutional Animal Care and Use Committee of the Children’s Hospital of Soochow University. The program was approved by the Medical Ethics Committee of Soochow University Children’s Hospital. Adequate measures were taken to minimize pain and the number of animals used. One hundred forty-five 27-day-old SD rats were randomly divided into two groups, a control group (*n* = 36) and a model group (*n* = 109). The control group was randomly divided into 3 subgroups with 12 rats each: normal zinc control group (Control group, 44 mg/kg Zn), Zn-deficient control group (ZD group, 2.7 mg/kg Zn), Zn supplemented control group (ZS group, 246 mg/kg Zn). The remaining 109 rats were used for the seizure model. Rats with successful modeling were randomly assigned to a subgroup of the seizure groups: pilocarpine-induced seizure plus regular zinc diet group (SE group, 44 mg/kg Zn), seizure plus low zinc diet group (SE + ZD group, 2.7 mg/kg Zn), seizure plus high zinc diet group (SE + ZS group, 246 mg/kg Zn).

### Induction of Status Epilepticus (Torolira et al., 2016)

Rats in each seizure group were injected with lithium chloride (127 mg/kg, i.p.) at P27. Twenty-four hours later (P28), scopolamine methyl chloride hydrobromide (1 mg/kg, i.p.) was injected to reduce peripheral cholinergic response. Thirty minutes later, pilocarpine (320 mg/kg, i.p.) was injected. Then, behavioral changes were observed. According to Racine classification (Racine, 1972), the model is considered to be successful and included in the study if the seizure degree reaches level IV or above; otherwise, the animal is excluded. Seventy-nine rats achieved grade IV and above in our experiments. Eleven animals died due to generalized tonic seizures, and the remaining 68 were randomly assigned to the SE group (*n* = 23), the SE + ZD group (*n* = 23), and the SE + ZS group (*n* = 22). During the subsequent feeding process, 3, 2, and 2 rats died in the SE, SE + ZD, and SE + ZS groups, respectively. In the six groups (Control, ZD, ZS, SE, SE + ZD, and SE + ZS), 12, 12, 12, 20, 21, and 20 rats survived, respectively. In each group, 10 rats (picric acid marker) were randomly labeled for weight measurement and behavioral testing. The remaining animals in each group were reserved for proteomics testing and were not included in the present study.

### Dietary Intervention

During the modeling period (P27, P28), each group was fed a normal zinc diet. After convulsion, Control and SE groups continued to receive a normal zinc diet (44 mg/kg zinc) for 28 days. ZD and SE + ZD groups were fed a low zinc diet (2.7 mg/kg zinc) for 28 days. ZS and SE + ZS groups were fed a high zinc diet (246 mg/kg zinc) for 28 days.

### Weight Monitoring

The body weight of rats in each group (labeling with picric acid) was recorded every 6 days.

### Novel Object Recognition Test (Jung et al., 2019)

Each rat was placed into a 50 cm × 50 cm × 50 cm uncovered test chamber for 10 min the day before the test as an introduction to the environment.

During the training phase, two identical objects are placed in two opposite parallel corners of the box, allowing each rat to probe both objects for 10 min.

For the test phase, one of the objects was replaced by another new object after a 1 h interval, and rats were allowed to explore again for 10 min. Exploring is defined as pointing the nose at an object and/or contacting the object with the nose at a distance of no more than 2 cm. Experiments were performed in a quiet environment to the extent that was possible, and the test chamber was wiped clean with 75% ethanol after each experiment. Rats have a natural tendency to explore novelty and usually spend more time exploring new objects than familiar ones that are remembered. The time that the rat spends exploring familiar objects and novel objects was recorded, and cognitive index was calculated (the time spent exploring the novel object divided by total exploration time).

### Determination of Serum Zinc Concentration

Blood samples were collected from the tail vein of mice in each group at P57. Blood samples were centrifuged at 3000 r/min for 10 min, and supernatants were transferred into labeled EP tubes. According to the instructions of the zinc assay kit, 12 μL sample (serum, with zinc standard solution and deionized water used as blanks), and 200 μL reagent I was added to a 37°C water bath for 5 min. Absorbance A1 was read at 570 nm, and then reagent II was added to a 37°C water bath for 5 min. Absorbance A1 was read at 570 nm, and absorbance A2 was read after adding reagent II in a water bath for 5 min. ΔA = A2–A1. Sample concentration = (ΔA sample)/(ΔA standard) ^∗^ Standard concentration.

### Establishment of a “Two-Hit” Model and Determination of Seizure Threshold (Ni et al., 2018)

After lithium chloride-pilocarpine induced seizures, animals from all six groups were injected with penicillin (5.1 × 106 U/kg/d, i.p.) at P57. The time to the first seizure after penicillin injection was recorded as seizure latency (min) (seizure threshold). The observation time was 1 h.

### Timm Staining

At the end of the seizure threshold test on P57, four rats from each group were randomly selected and each given an i.p. injection of chloral hydrate at a dose of 1 ml/100 g followed by regular Timm staining (*n* = 4/each group, P57) (Ni et al., 2009). Briefly, animals were perfused through the heart with 0.4% sodium sulfide (100 ml) and then 4% PFA in PBS (100 ml). The brains were equilibrated sequentially with 30% sucrose at 4°C for further analysis. 30 μm-thick coronal brain sections were stained in the solutions consisted of 30% gum arabic, 3.825% citric acid, 3.525% sodium citrate, 3.525% hydroquinone, and 25.5% silver nitrate. The slides were incubated at 26°C for 70 min. The person who scored the Timm staining was blind to the experimental grouping.

### Western Blot Analysis

The remaining rats in each group were perfused with 4% chloral hydrate (1 ml/100 g, P57). Hippocampi were placed in a precooled labeled EP tube and then quickly stored at −80°C. Four rat tissues were taken from each group for protein extraction, and the remaining tissues were frozen for other purposes. Detailed steps of the Western blotting method have been previously described (Ni et al., 2018).

Briefly, polyvinylidene fluoride membrane blots were incubated with one of the following antibodies: goat anti-ZnT3 (1:1000, Santa Cruz), rabbit anti-GPR39 polyclonal antibody (1:1000, Biorbyt), rat anti- MBP monoclonal antibody (1:2000, Abcam), mouse anti-GAPDH monoclonal antibody (1:5000, Proteintech) or rabbit anti-β-tubulin polyclonal antibody (1:5000, Proteintech) in TBST contain 5% non-fat dry milk overnight at 4°C. Membranes were then incubated with secondary antibodies for approximately 2 h. ECL chemiluminescence kit A and B were mixed in equal volume, and PVDF membranes were immersed in the luminescent liquid for approximately 2 min, and then the film was placed in an automatic developing device for exposure (LAS 4010, GE Healthcare Life Sciences, Little Chalfont, United Kingdom). Grayscale values of each band were analyzed using ImageJ image processing software.

### Statistical Analysis

Body weight was analyzed by two-way, repeated-measures ANOVA (treatment as a between subject factor and training day as a within subject factor). Serum zinc concentration, seizure threshold, Timm staining, neurobehavioral data and protein levels were analyzed using one-way ANOVA with *post hoc* tests. Data are presented as the mean ± SD. Statistical significance was considered *P* < 0.05.

## Results

### Weight Monitoring

**FIGURE 1:**
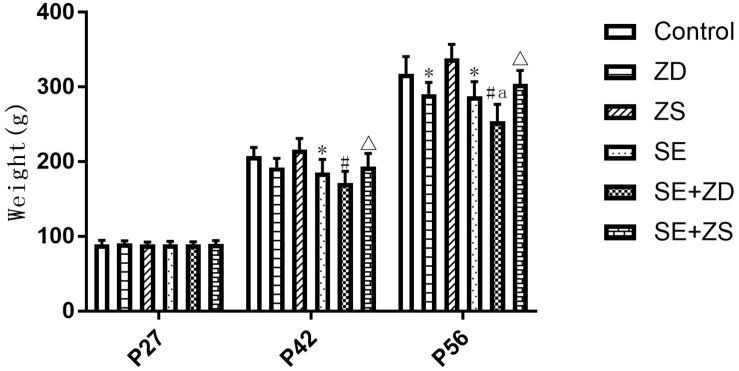
Comparison of body weights at different ages (g, *n* = 10/group). There were no significant differences between rats in each group at P27. At P42, the body weight of lithium chloride-pilocarpine-treated injured rats of SE, SE + ZD, and SE + ZS groups was significantly decreased compared to Control, ZD and ZS groups, respectively. At P56, the body weight of lithium chloride-pilocarpine-treated rats of SE, SE + ZD, SE + ZS, ZD, and SE + ZD groups was significantly decreased compared to Control, ZD, ZS, Control, and SE groups, respectively (two-way repeated-measures ANOVA, ^∗^*P* < 0.05, compared to Control group, #*P* < 0.05 compared to ZD group, Δ*P* < 0.05 compared to ZS group, a *P* < 0.05 compared to SE group).

As shown in Figure 1, during establishment of the seizure model (P27), there were no significant differences in body weight between rats in each group. At P42, the body weight of SE, SE + ZD, and SE + ZS groups was statistically decreased compared to Control, ZD and ZS groups, respectively (*P* < 0.05). At P56, the body weight of SE, SE + ZD, SE + ZS, ZD, and SE + ZD groups was significantly decreased compared to Control, ZD, ZS, Control, and SE groups, respectively (*P* < 0.05).

### Novel Object Recognition Test

**FIGURE 2:**
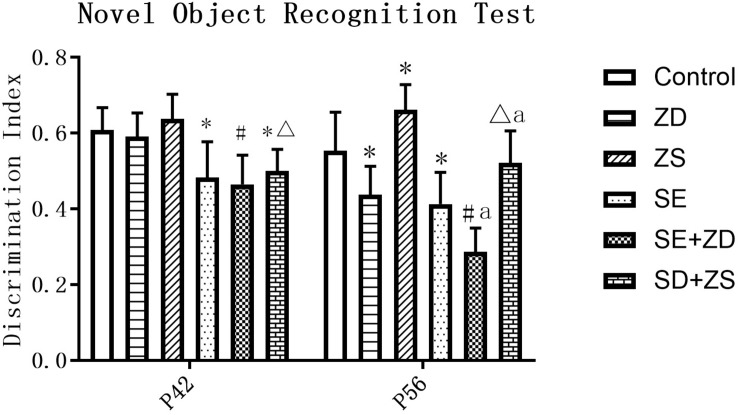
Novel object recognition test. At P42, the recognition indexes were significantly reduced in the pilocarpine-treated rats of SE, SE + ZD, and SE + ZS groups compared to the corresponding control, ZD, ZS, and control groups, respectively. At P56, the recognition indexes were significantly reduced in SE, SE + ZD, SE + ZS, ZD, and SE + ZD groups compared to the corresponding control, ZD, ZS, control and SE groups, respectively (one-way ANOVA with *post hoc* tests, ^∗^*P* < 0.05, compared to Control group, #*P* < 0.05 compared to ZD group, Δ*P* < 0.05 compared to ZS group, a *P* < 0.05 compared to SE group *n* = 10/group).

At P42, there was a significant difference in the recognition index of each group (*P* < 0.05). Further pairwise comparison revealed that recognition indexes were statistically reduced in SE, SE + ZD, and SE + ZS groups compared to the corresponding control, ZD and ZS groups, respectively (*P* < 0.05). At P56, there was a significant difference in the recognition index of each group (*P* < 0.05). Further pairwise comparison indicated that recognition indexes were statistically reduced in SE, SE + ZD, SE + ZS, ZD, and SE + ZD groups compared to the corresponding control, ZD, ZS, control, and SE groups, respectively (*P* < 0.05, Figure 2).

### Determination of Serum Zinc Concentration

**FIGURE 3:**
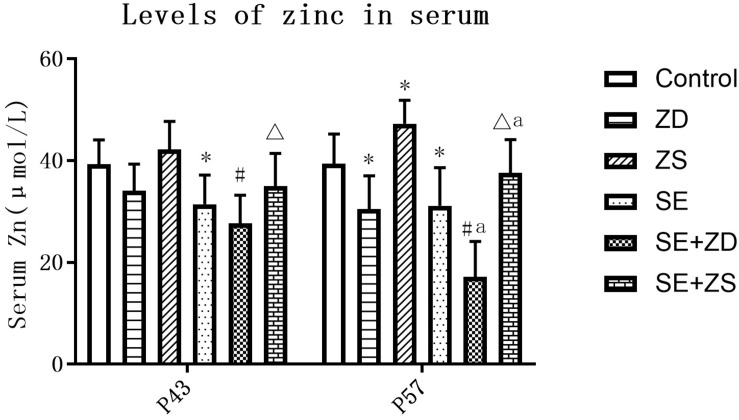
Determination of serum zinc concentration (μmol/L). At P43, serum zinc concentrations of rats in the pilocarpine-treated SE, SE + ZD, and SE + ZS groups were significantly lower than corresponding groups of Control, ZD and ZS groups, respectively. At P57, there were significantly lower serum zinc concentrations in SE, SE + ZD, SE + ZS, ZD, and SE + ZD groups compared to corresponding Control, ZD, ZS, Control, and SE groups, respectively. In addition, at P57, serum zinc concentrations in ZS and SE + ZS groups were significantly increased compared to corresponding Control and SE groups, respectively (one-way ANOVA with *post hoc* tests, ^∗^*P* < 0.05, compared to Control group, #*P* < 0.05 compared to ZD group, Δ*P* < 0.05 to ZS group, a *P* < 0.05 compared to SE group).

There were statistical differences in serum zinc concentration among the six groups at both P43 and P57 (*P* < 0.05). At P43, serum zinc concentrations in the SE, SE + ZD, and SE + ZS groups were obviously lower than corresponding groups of Control, ZD, and ZS groups, respectively (*P* < 0.05). At P57, there were significantly lower serum zinc concentrations in SE, SE + ZD, SE + ZS, ZD, and SE + ZD groups compared to corresponding Control, ZD, ZS, Control, and SE groups, respectively (*P* < 0.05). In addition, at P57, serum zinc concentrations in ZS and SE + ZS groups were statistically increased compared to corresponding Control and SE groups, respectively (*P* < 0.05, Figure 3).

### Seizure Threshold

**FIGURE 4:**
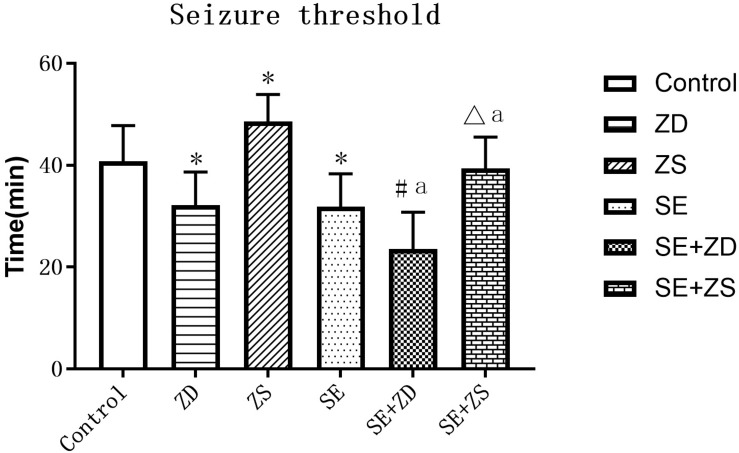
Seizure threshold analysis (min). There were significantly decreased seizure thresholds in rats of SE, SE + ZD, SE + ZS, ZD, Control, SE + ZD, and SE groups compared with corresponding Control, ZD, ZS, Control, ZS, SE, and SE + ZS groups, respectively (one-way ANOVA with *post hoc* tests, ^∗^*P* < 0.05, compared to Control group, #*P* < 0.05 compared to ZD group, Δ*P* < 0.05 compared to ZS group, a *P* < 0.05 compared to SE group).

We compared seizure susceptibility by exposing the rats to penicillin. The time to the first seizure after penicillin injection was recorded as the seizure latency (min) (seizure threshold). Seizure onset is obviously earlier in penicillin-treated rats in SE, SE + ZD, SE + ZS groups than in non-penicillin-treated rats in Control, ZD, ZS groups, respectively. In addition, there were strongly decreased seizure thresholds in ZD and SE + ZD groups compared with corresponding Control and SE groups, respectively. Furthermore, there were significantly increased seizure thresholds in ZS and SE + ZS groups compared with corresponding Control and SE groups, respectively (*P* < 0.05, Figure 4).

### Timm Staining

**FIGURE 5:**
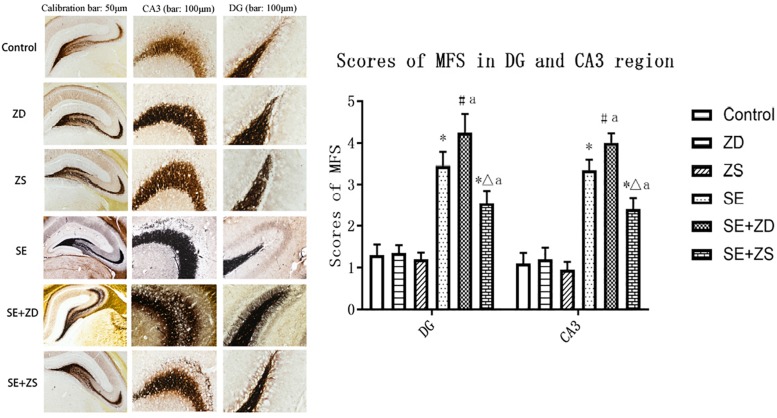
Mossy fiber sprouting in CA3 and dentate gyrus subfields by Timm staining. Developmental seizures significantly increased the Timm particle scores in the hippocampal CA3 and DG subfields of rats in SE, SE + ZD, and SE + ZS groups compared to corresponding Control, ZD and ZS groups, respectively. Moreover, the Timm particle scores were significantly higher in SE + ZD group compared to the SE group. Further, Timm particle scores were significantly lower in the SE + ZS group than in the SE group (one-way ANOVA with *post hoc* tests, ^∗^*P* < 0.05, compared to Control group, #*P* < 0.05 compared to ZD group, Δ*P* < 0.05 compared to ZS group, a *P* < 0.05 compared to SE group, *n* = 4/group).

There were significant differences in the sprouting scores of mossy fibers in the hippocampal CA3 and DG subfields among the six groups (*P* < 0.05). The Timm particle scores in the hippocampal CA3 and DG subfields were obviously increased in SE, SE + ZD, and SE + ZS groups compared to corresponding Control, ZD, and ZS groups, respectively (*P* < 0.05). Compared to the SE group, Timm particle scores in hippocampal CA3 and DG areas of the SE + ZD group were significantly increased (*P* < 0.05). Timm particle scores of hippocampal CA3 and DG in the SE + ZS group were still higher than in Control, while scores were lower than in the SE group (*P* < 0.05). There were no significant differences in Timm particle scores between ZD and Control groups or between ZS and Control groups (Figure 5).

### Western Blot Analysis

**FIGURE 6:**
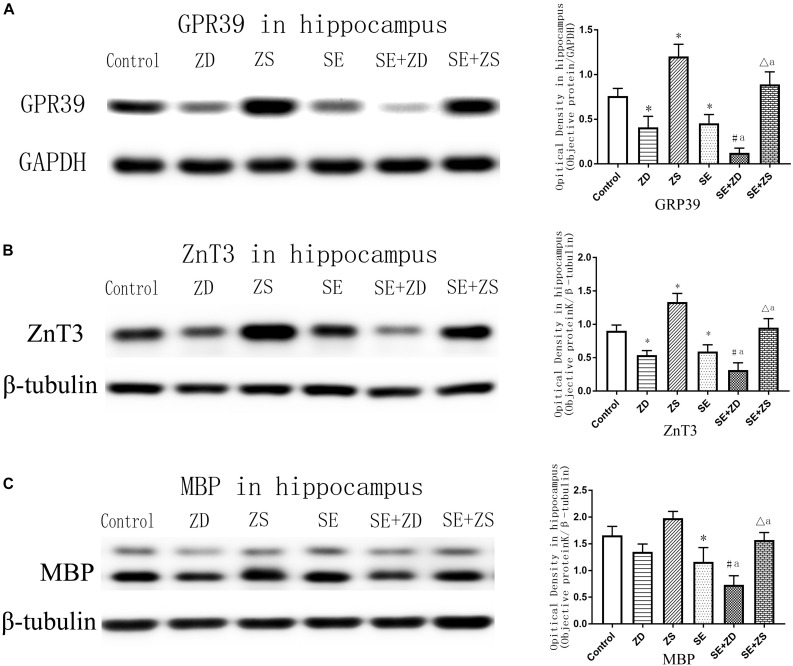
Protein levels of GPR39 **(A)**, ZnT-3 **(B)**, and MBP **(C)** in the hippocampus (one-way ANOVA with *post hoc* tests, ^∗^*P* < 0.05, compared to Control group, #*P* < 0.05 compared to ZD group, Δ*P* < 0.05 compared to ZS group, a *P* < 0.05 compared to SE group, *n* = 4/group).

As shown in Figure 6A (GPR39) and Figure 6B (ZnT3), expression of GPR39 and ZnT3 were markedly lower in ZD and SE groups compared to the control group (*P* < 0.05). In contrast, expression of GPR39 and ZnT3 were significantly higher in the ZS group than in the control group (*P* < 0.05). Expression of GPR39 and ZnT3 were obviously lower in the SE + ZD group compared to the ZD and SE groups (*P* < 0.05). Furthermore, expression of GPR39 and ZnT3 were significantly upregulated in the SE + ZS group compared to the SE group but were obviously decreased compared to the ZS group (*P* < 0.05).

There was markedly downregulated expression of MBP in penicillin-treated SE, SE + ZD, and SE + ZS groups compared to corresponding control, ZD and ZS groups, respectively. Compared with the SE group, MBP expression was further decreased in the SE + ZD group (*P* < 0.05), and compared to the SE group, MBP expression was significantly increased in the SE + ZS group (*P* < 0.05) (Figure 6C).

## Discussion

This study investigated the effects of different concentrations of dietary zinc on long-term cognition, seizure threshold, MF sprouting and gene expression in the hippocampus following lithium chloride-pilocarpine-induced developmental seizures. There are three main findings in this study. First, a zinc-deficient diet for 4 weeks aggravates seizure-induced weight loss, cognitive impairment and decreased concentration of serum zinc as well as seizure threshold, while increasing aberrant mossy fiber sprouting, which were in parallel with reduced expression of GPR39, ZnT -3 and MBP in the hippocampus. Second, a zinc supplemented diet for 4 weeks significantly improves the abovementioned injury-related changes caused by developmental seizures and may correct the abnormal expression of GPR39, ZnT-3, and MBP in the hippocampus. Third, the expression pattern of MBP in hippocampus coincided with aberrant sprouting of mossy fibers. In addition, low-zinc or high-zinc diet intervention for 2 weeks exerted no significant effect on body weight, cognition, or serum zinc concentrations.

Epidemiological data indicate that a large number of diseases affecting one-third of the world’s population is attributed to zinc deficiency, and a lack of bioavailable zinc intake in the diet is the most common cause of zinc deficiency (Abbaspour et al., 2015). Karweina et al. (2015) showed that high doses of zinc oxide may lead to imbalanced zinc homeostasis in animals and changes in ZIP4 methylation. Zinc deficiency and zinc supplementation also affect homocysteine levels and related enzyme expression in rats (Jing et al., 2015). Rats fed rice with fortified zinc oxide exhibited significantly increased body weight (Della Lucia et al., 2014). Our current results are consistent with these studies. Herein, three different dietary Zn levels were used in growing rats according to previous studies (Nagatomo et al., 1998; Takeda et al., 2006b). Serum zinc concentrations were measured at P43 and P57. Results showed that at P57 following 4 weeks of differential diet treatments, there were significantly lower serum zinc concentration in the ZD group and significantly higher serum zinc concentration in ZS groups compared to the control group. Correspondingly, low-zinc and high-zinc diet interventions for 4 weeks had significant effects on body weight and cognition in control groups. These results indicate that long-term zinc deficiency or high zinc in the diet causes decreased or increased blood zinc concentrations, potentially affecting growth and development. However, our study also showed that different doses of zinc in the diet for 2 weeks had no significant effects on body weight or cognition in normal rats and showed no significant differences in serum zinc concentration in ZD and ZS groups compared to the normal zinc diet control group at P43. This indicates that under normal physiological conditions, the body has a regulatory mechanism for blood zinc concentration, which is not easily imbalanced by dietary zinc deficiency.

There are many studies on the effects of zinc loading or deprivation on seizure thresholds. The earliest report came from the Barry-Sterman team. They found that zinc loading delayed kindled seizure induction, while zinc deprivation accelerated kindling (Barry-Sterman et al., 1986). Subsequently, using seizure-susceptible EL mice, Fukahori and Itoh (1990) found that zinc deficiency causes increased seizure susceptibility, while reducing by zinc loading, but an adequate diet had no effect. However, using the same epilepsy-susceptible EL mice, a study by Nagatomo et al. (1998) denied this conclusion. They showed that convulsive seizures in EL mice fed a Zn-deficient diet for 4 weeks were more effectively inhibited than in mice receiving a high zinc or sufficient zinc diet for the same period. However, later studies by Takeda et al. (2003a, 2005, 2006a, 2009) reinforced the idea that young mice fed a zinc-deficient diet for 4 weeks experienced reduced convulsive threshold. This is in accordance with our present findings. Herein, we also found that zinc-deficient diets for 4 weeks reduced seizure threshold, while the threshold increased after 4 weeks of a zinc supplemented diet. The underlying mechanisms whereby this occurs have not been clearly defined, but parallel changes in cognitive deficits and aberrant sprouting of hippocampal mossy fibers provide valuable clues.

Here, we compared the different effects of zinc loading or deprivation on cognitive function through the novel object recognition test. The novel object recognition test is a popular method for detecting rodent non-spatial memory neurobiology. The principle of this test is that rodents have a natural tendency to explore new items. The memory capacity and degree of damage are assessed by quantitatively calculating the amount of time the rodent spends exploring presented objects. Here, we showed that at P42 following 2 weeks zinc loading or deprivation, there was no significant difference in the recognition index of each group among the non-penicillin-treated control, ZD and ZS groups. On the other hand, however, the recognition indexes were significantly reduced in penicillin-treated SE, SE + ZD, and SE + ZS groups compared to the corresponding control, ZD and ZS groups, respectively. This suggests that developmental seizures can cause long-term cognitive impairment, which is consistent with previous studies (Ni et al., 2009, 2018). The results also showed that 2-week dietary treatment with different zinc concentrations had no significant intervention effect on cognitive impairment. Notably, at P56 following 4 weeks zinc loading or deprivation, although there was still a significant reduction in the recognition index of penicillin-treated SE and SE + ZD groups compared to the corresponding control and ZD groups, but the recognition index in rats of the zinc loading-treated SE + ZS group increased significantly compared to the normal diet-treated SE group, which indicated that the 4-week zinc loading diet can significantly improve long-term cognitive impairment caused by developmental seizures. We hypothesize that this neuroprotective effect of zinc loading diet may be related to the regulation of hippocampal zinc metabolism homeostasis. The rodent hippocampus plays an important role in the memory of the encoded object, and temporary or permanent damage to the hippocampus will destroy the memory of the object (Cohen and Stackman, 2015). The hippocampus is susceptible to zinc deficiency in the brain. In particular, zinc deficiency reduces the reactive zinc pool, as revealed by hippocampal histochemical Timm staining, which is closely related to high susceptibility to seizures (Takeda et al., 2003b, 2006b; Takeda and Tamano, 2009). Zn(2+) coexists with glutamate at the end of hippocampal mossy fiber and regulates its release, thereby affecting synaptic function (Besser et al., 2009). The incidence of tonic-clonic convulsion was markedly increased after i.p. injection of zinc chelators Clioquinol (CQ) and TPEN in adult Noda epileptic rats (Takeda et al., 2013). Combined with the finding in this study that zinc loading diet intervention markedly improved the regenerative mossy fiber sprouting in hippocampus induced by developmental seizures as revealed by Timm staining, we speculate that one of the mechanisms for this neuroprotection may be related to the improvement of zinc transporter-mediated mossy fiber spouting in hippocampus.

In this study, we examined expression of zinc metabolism-related genes. GPR39 is a rhodopsin-like G protein-coupled receptor consisting of 435 amino acid residues and is classified as a member of the ghrelin/neurotensin receptor subfamily according to its amino acid sequence. In addition to its expression in the gastrointestinal tract, liver, fat, and pancreas, GPR39 is also widely expressed in the hippocampus, which is also a zinc-enriched region, suggesting the neurotransmitter role of zinc may be related to GPR39 (Khan, 2016). It was initially thought that obestatin was a natural ligand for GPR39, and later Holst et al. (2007) suggested that Zn2+ may be a “physiological regulator” of GPR39. Synaptically released Zn(2+) triggers metabotropic activity in the CA3 region of cultured hippocampal neurons, which was attenuated by knockdown of GPR39 expression (Besser et al., 2009). Our present findings indicate that a zinc-deficient diet can further lead to decreased expression of GPR39 in the hippocampus, which in turn aggravates cognitive impairment. In contrast, we demonstrated for the first time that a zinc supplemented diet significantly improves cognitive dysfunction and up-regulates expression of GPR39 in the hippocampus, suggesting a protective effect of high-zinc intervention on developmental seizure-induced brain damage. In combination with clinical studies, which indicate that serum zinc levels in untreated epileptic children and in children with systemic refractory epilepsy were significantly lower than in healthy controls (Wojciak et al., 2007; Seven et al., 2013; Kheradmand et al., 2014; Saad et al., 2014), our results suggest that a zinc supplemented diet may have potential translational medical value for the intervention of developmental convulsive brain damage.

Currently, only two studies from Siegward and Kumar have found that a zinc supplemented diet suppresses convulsive seizures and reduce seizure thresholds (Elsas et al., 2009; Kumar et al., 2015). In addition, a study by Contestabile et al. (2016) found that 4 weeks of zinc supplementation strongly impairs consolidation of hippocampal-dependent memory in wild type adult rats through contextual fear conditioning and inhibitory avoidance testing. This is consistent with our present results. Herein, we also showed that the recognition indexes were significantly reduced in ZD groups compared to the control group. Moreover, our study demonstrated that a zinc supplemented diet attenuates cognitive impairment caused by developmental seizures, as shown by the comparison between SE and SE + ZS groups. This is in accordance with a study from Cope et al. (2011) who revealed that supplemental zinc prevents cognitive and behavioral deficits associated with traumatic brain injury (TBI).

Of particular interest, we found that the intervention effect of a zinc supplemented diet on cognitive impairment caused by developmental seizures was consistent with the effect on hippocampal regenerative mossy fiber sprouting and was consistent with expression of MBP. The mechanism for this phenomenon is still unclear. However, the finding that hippocampal regenerative mossy fiber sprouting and MBP are both brain damage indicators rather than the cause of brain damage may help explain the phenomenon of synchronization between the two indicators in the pathophysiological process of brain injury and repair. MBP is one of the most abundant structural proteins in oligodendrocyte myelin and is essential for cognitive function. MBP is negatively correlated with the severity of seizure-induced brain injury and has been recognized as a biomarker for brain damage. MBP is reduced in epileptic foci in patients with refractory epilepsy, suggesting that this change interferes with the transmission of nerve impulses (Marchi et al., 2010). Mesial temporal lobe epilepsy (MTLE) is usually associated with cognitive deficits. Proteomics analysis by Persike et al. (2018) demonstrated up-regulated protein level of MBP in MTLE patients, suggesting a compensatory mechanism due to epilepsy-related nerve damage. Song et al. (2018) observed the damage of myelin microstructure and the decrease of MBP caused by lithium-pilocarpine-induced state convulsion in immature rats by transmission electron microscopy and Western blot analysis, this damage was improved after down-regulating Lingo-1 expression. Meanwhile, it is recently noted that the seizure-induced hippocampal mossy fiber sprouting may not be a potential therapeutic target for epilepsy, but only a pathological hallmark change caused by convulsive brain injury (Cavarsan et al., 2018; Koyama and Ikegaya, 2018). Further, MBP is also reported to induce the proliferation of cultured Schwann cells and astrocytes and thus improve the integrity of the neuronal plasma membrane (Tzeng et al., 1999; Snaidero et al., 2017). Based on these findings, we speculate that similar alterations between the expression pattern of MBP and aberrant sprouting of mossy fibers in the hippocampus may indicate that sprouting is a secondary pathological change caused by developmental brain damage rather than the cause of epileptogenesis. Up-regulation of MBP protein levels in the high zinc diet-treated seizure group (SE + ZS), as well as the corresponding improvement of cognitive impairment and reduced hippocampal mossy fiber regenerative sprouting, may represent a compensatory mechanism for neuronal membrane damage and repair.

It is worth noting that expression patterns of ZnT-3 observed in this study were not entirely consistent with observed changes in hippocampal mossy fiber sprouting. However, this does not negate the traditional notion that ZnT-3 is a sprouting marker. Since we tested expression levels of ZnT-3 protein in the entire hippocampus, we did not selectively detect expression of ZnT-3 in the mossy fiber pathway. Local anatomic-specific pathway expression changes using immunohistochemistry techniques merit further investigation.

## Conclusion

A Zinc-deficient diet for 4 weeks aggravates long-term adverse effects of developmental seizures, including parameters of weight, cognition, seizure threshold and serum zinc concentration, which paralleled expression changes observed in hippocampal GPR39 and ZnT-3. In contrast, zinc supplementation for 4 weeks significantly improved the above described damage-related changes, attenuating the abnormal expression of GPR39, ZnT-3, and MBP in the hippocampus. Similar alterations between the expression pattern of MBP and aberrant sprouting of mossy fibers in the hippocampus may indicate that sprouting is a secondary pathological change caused by developmental brain damage rather than the cause of epileptogenesis. Up-regulation of MBP protein levels in the high zinc diet-treated seizure group (SE + ZS), as well as the corresponding improvement of cognitive impairment and reduced hippocampal mossy fiber regenerative sprouting, may represent a compensatory mechanism for neuronal membrane damage and repair.

## Data Availability

All datasets generated for this study are included in the manuscript and/or the supplementary files.

## Ethics Statement

Animal Subjects: The animal study was reviewed and approved by the Institutional Animal Care and Use Committee of the Children’s Hospital of Soochow University. The program was approved by the Medical Ethics Committee of Soochow University Children’s Hospital.

## Author Contributions

HN designed the study, analyzed the data, and wrote the manuscript. N-NC, D-JZ, Y-XS, and D-DW were the operators of the experiment and were responsible for the statistical analysis of data.

## Conflict of Interest Statement

The authors declare that the research was conducted in the absence of any commercial or financial relationships that could be construed as a potential conflict of interest.
